# Supplementary material for: Electrochemical Sensing of Pb2+ and Cd2+ Ions with the Use of Electrode Modified with Carbon-Covered Halloysite and Carbon Nanotubes
Source: Molecules. 2022 Jul 19;27(14):4608. doi: 10.3390/molecules27144608 (PMC9324300; doi:10.3390/molecules27144608)
Supplement: Supplementary file 1 [file molecules-27-04608-s001.zip › molecules-1779449-supplementary.pdf]

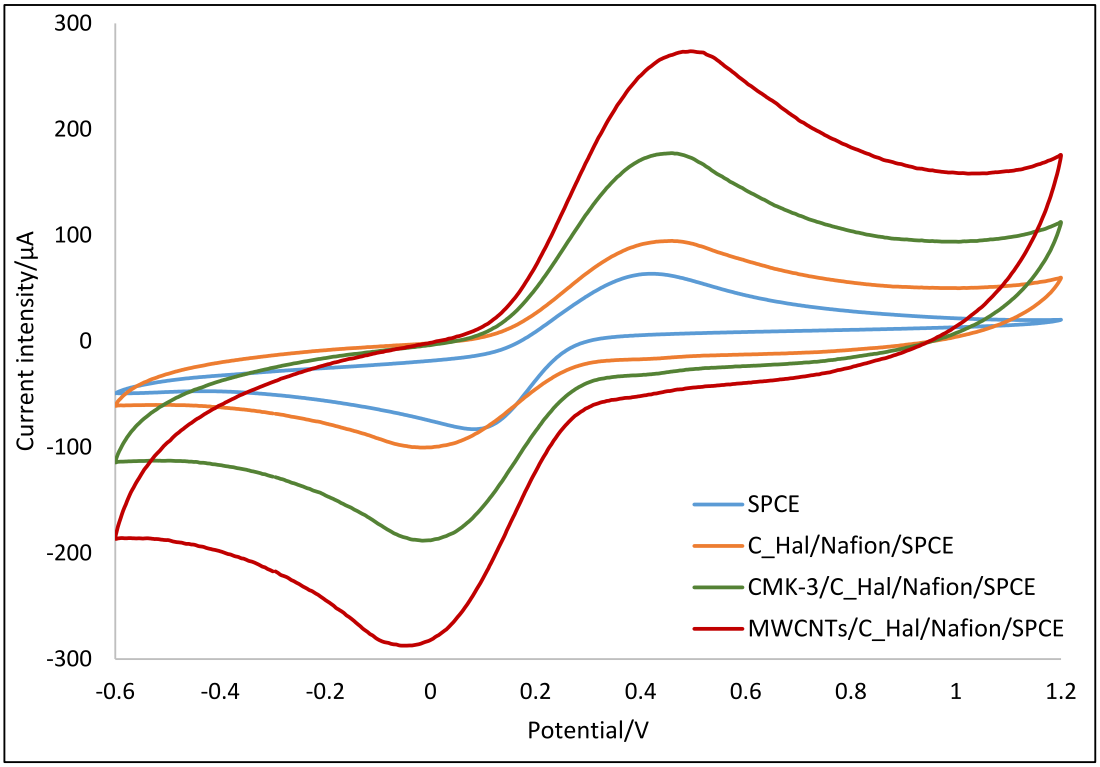

(**A**)

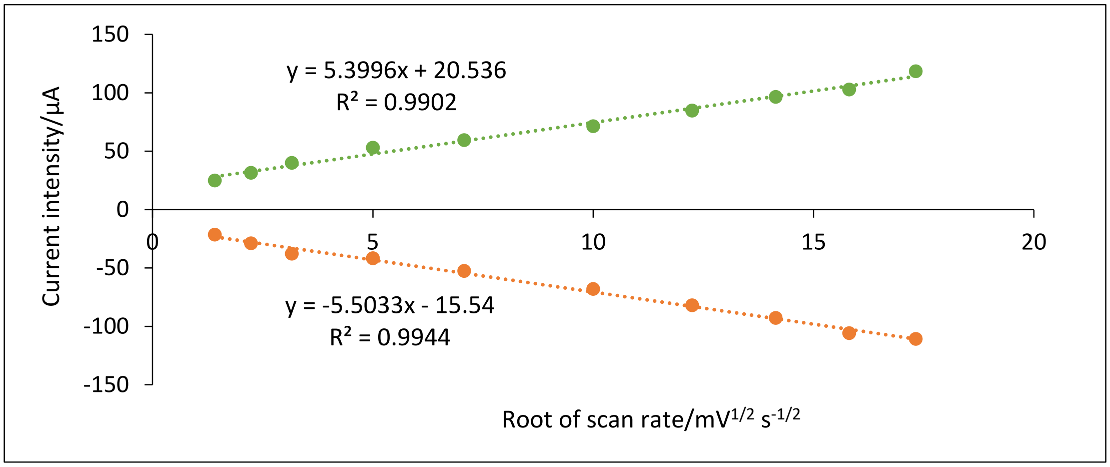

(**B**)

Figure S1. (**A**) Cyclic voltammograms recorded in the presence of 1 mmol L^{-1} $[Fe(CN)_6]^{3-/4-}$ in 0.1 mol L^{-1} KCl using SPCE (blue line), C_Hal/Nafion/SPCE (orange line), CMK-3/C_Hal/Nafion/SPCE (green line) and MWCNTs/C_Hal/Nafion/SPCE (red line), scan rate = 100 mV s^{-1}.(**B**). The dependence of the intensity of the current of the oxidation (green points) and reduction (orange points) peaks on the root of scan rate of the working electrode MWCNTs/C_Hal/Nafion/SPCE.

Table S1. Coefficients of the equation of the response surface (SE - standard error).

Optimization of the Sensitivity of the Cadmium Determination Method				Optimization of the Sensitivity of the Lead Determination Method			
Coeff.	Value	SE	p-Value	Coeff.	Value	SE	p-Value
b0	1.38	2.37	0.57849	b0	21.46	8.56	0.04061
b1	6.28	2.97	0.07268	b1	4.53	10.73	0.68582
b2	11.86	2.97	0.00528	b2	33.01	10.73	0.01793
b3	−5.94	1.22	0.00185	b3	−15.47	4.42	0.00996
b4	5.02	1.59	0.01606	b4	20.86	5.74	0.00836
b5	−7.23	1.22	0.00060	b5	−25.00	4.42	0.00077

Table S2. Optimization of DPASV parameters using simplex method for determination of Cd(II) and Pb(II) ions.

Point	Parameter				Cd		Pb	
	Es [mV]	Ep [mV]	tp [ms]	υ [mV s^{-1}]	Sensitivity [µA·L·µmol^{-1}]	R^2	Sensitivity [µA·L·µmol^{-1}]	R^2
P1	10	50	10	10	0.042	0.999	0.437	0.924
P2	20	50	10	10	0.030	0.989	0.050	0.958
P3	15	94	10	10	0.057	0.992	0.120	0.892
P4	15	65	20	10	0.097	0.967	0.120	0.882
P5	15	65	12	18	0.047	0.994	0.080	0.964
P6	7.5	86	15	14	0.110	0.975	0.140	0.838
P7	1.5	104	18	16	0.130	0.992	0.307	0.922
P8	13	113	19	17	0.177	0.953	0.264	0.905
P9	15	145	23	20	0.205	0.996	0.385	0.870
P10	16	177	28	24	0.222	0.988	0.548	0.883
P11	18	208	32.3	27	0.256	0.988	0.823	0.895
P12	9.5	171	27	14	0.223	0.973	0.495	0.861
P13	7	180	38	24	0.190	0.823	0.434	0.957
P14	9	166	29	20	0.159	0.992	0.794	0.772
P15	20	258	45	26	0.317	0.988	0.842	0.979
P16	15	150	25	15	0.338	0.997	1.007	0.990
P17	29	240	25	19	0.264	0.948	0.684	0.883
P18	14	200	33	22	0.244	0.994	0.633	0.941
P19	17	215	33	22	0.231	0.980	0691	0873
P20	19	226	35	24	0.328	0.994	0.805	0.927
P21	20	222	33	25	0.325	0.992	0.777	0.967

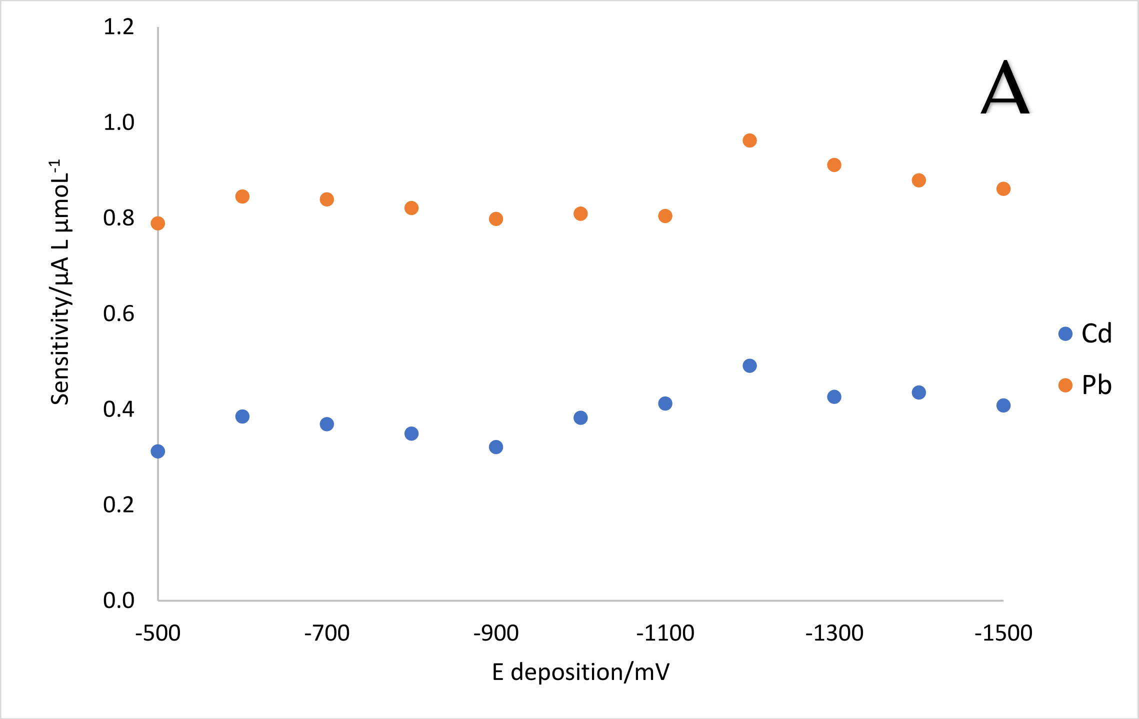

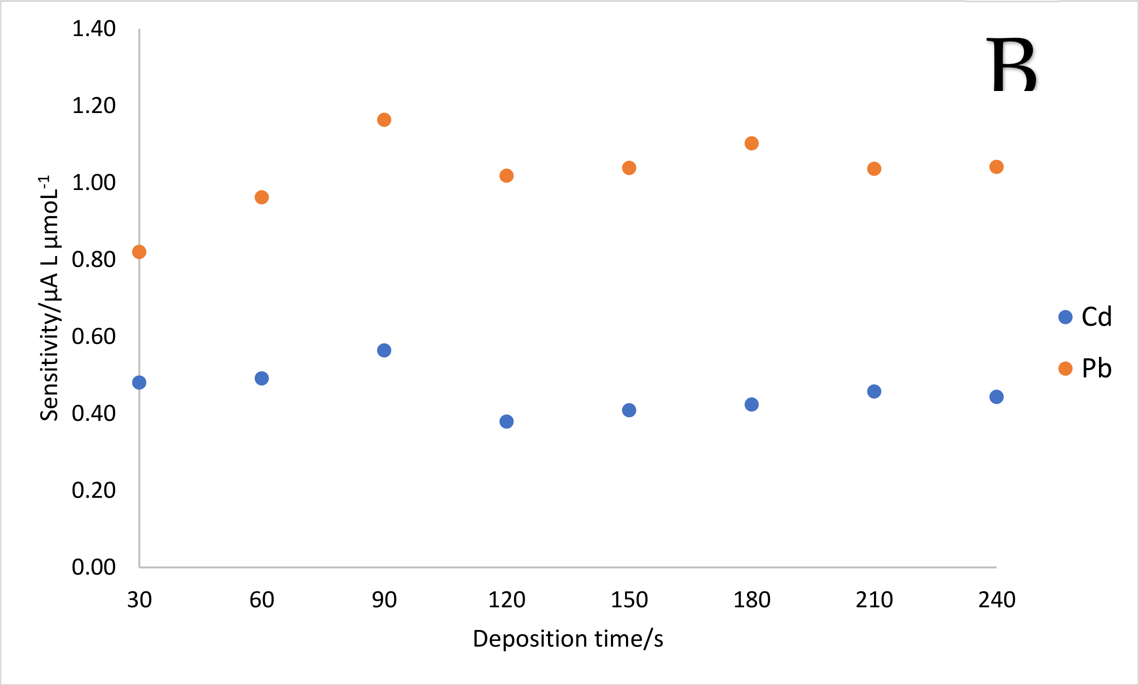

Figure S2. The dependence of sensitivities obtained for both analytes on the potential (**A**) and time (**B**) of deposition.
